# The role of growth factor receptors in viral infections: An opportunity for drug repurposing against emerging viral diseases such as COVID‐19?

**DOI:** 10.1096/fba.2020-00015

**Published:** 2020-04-11

**Authors:** Hubert Hondermarck, Nathan W. Bartlett, Victor Nurcombe

**Affiliations:** ^1^ School of Biomedical Sciences and Pharmacy Faculty of Health and Medicine University of Newcastle Callaghan NSW Australia; ^2^ Hunter Medical Research Institute University of Newcastle New Lambton Heights NSW Australia; ^3^ Institute of Medical Biology Glycotherapeutics Group A*STAR Singapore; ^4^ Lee Kong Chian School of Medicine Nanyang Technology University‐Imperial College London Singapore

**Keywords:** cancer drugs, COVID‐19, growth factors, heparan sulfate, heparin, inhibitors, SARS‐CoV‐2, tyrosine kinase, virus

## Abstract

Growth factor receptors are known to be involved in the process of viral infection. Many viruses not only use growth factor receptors to physically attach to the cell surface and internalize, but also divert receptor tyrosine kinase signaling in order to replicate. Thus, repurposing drugs that have initially been developed to target growth factor receptors and their signaling in cancer may prove to be a fast track to effective therapies against emerging new viral infections, including the coronavirus disease 19 (COVID‐19).

**Abbreviations**
ACE2: angiotensin‐converting enzyme 2
COVID‐19: coronavirus disease 19
EBV: Epstein‐Barr virus
EGFR: epidermal growth factor receptor
FGFR: fibroblast growth factor receptor
GFRs: growth factor receptors
HBV: hepatitis B virus
HCV: hepatitis C virus
HER2: human epidermal growth factor receptor 2
HIV: human immunodeficiency virus
HS: heparan sulfates
mAbs: monoclonal antibodies
MERS‐CoV: middle east respiratory syndrome coronavirus
NGF: nerve growth factor
RBD: receptor binding domain
SARS‐CoV: severe acute respiratory syndrome coronavirus
SARS‐CoV‐2: severe acute respiratory syndrome coronavirus 2
TGFbR: transforming growth factor beta receptor
TMPRSS2: transmembrane protease, serine 2
VACV: vaccinia virus

## 1 INTRODUCTION

The recent and rapid worldwide spread of the severe acute respiratory syndrome coronavirus 2 (SARS‐CoV‐2), that gives rise to the coronavirus disease 19 (COVID‐19),^1^ points to the urgent need for therapies against emerging new viruses. As there is currently no vaccine nor effective antiviral therapy for SARS‐CoV‐2, innovative approaches need to be developed rapidly. The repurposing of existing drugs, which are currently used, or have been used, against other diseases, represents a potential fast track to effective clinical treatment. Large‐scale and hypothesis‐free drug screening against viral infections can be costly and time consuming and, therefore targeted strategies are more suitable. However, a major difficulty is often the limited amount of information about the molecular mechanisms involved in the pathogenicity of a virus when a new outbreak starts.

Viruses are cellular parasites that infect eukaryotic and prokaryotic cells and “hijack” their cellular machinery to replicate themselves before being released to further infect neighboring cells and eventually other organisms. The first step in viral infection is the attachment of the virus to the plasma membrane and its entry into the cell, which is followed by intracellular viral replication and finally the release of the newly formed viruses. With regard to COVID‐19, it is known that the angiotensin‐converting enzyme 2 (ACE2) ACE2 provides the cell membrane receptor entry point for SARS‐CoV‐2.^2^, ^3^, ^4^ The structure‐function relationships and antigenicity of the viral SARS‐CoV‐2 spike glycoprotein have also been established.^4^ The structural basis for the attachment of SARS‐CoV‐2 to the cell membrane is the binding of the receptor binding domain (RBD) of the surface spike glycoprotein (S protein) of SARS‐CoV‐2 to ACE2, as revealed by X‐ray crystallography.^5^ In addition, it has also been shown that SARS‐CoV‐2 uses the transmembrane protease serine 2 (TMPRSS2) for S protein priming (cleavage of the fusion domain) and the inhibition of TMPRSS2 by a clinically approved inhibitor might block further infection.^3^ Such narratives are currently developing; it is still early, and these initial findings obviously do not preclude the identification of other molecular partners involved in the entry and replication of SARS‐CoV‐2 in human cells.

Growth factor receptors (GFRs), are transmembrane proteins expressed in eukaryotic cells and whose primary function is to bind to extracellular polypeptide growth factors. The binding of specific growth factors to GFRs results in the activation of their intracellular protein kinase domain that initiates a cascade of signaling events ultimately leading to the regulation of cell growth. Interestingly, GFRs have also been identified as necessary for the entry of some viruses, including coronaviruses, and GFR signaling is involved in viral replication in many instances. Drugs targeting GFRs and their signaling are currently used in clinical practice for the treatment of cancer and given the role of GFRs in virus entry and replication, these drugs could potentially be repurposed against viral infections. In this article, the evidence for the link between viral infection and GFRs will be reviewed, and the value of repurposing current oncologic drugs targeting GFRs in order to expand effective antiviral strategies, including in the fight against COVID‐19, will be discussed.

## 2 ROLE OF GFRS IN CELLULAR GROWTH

In multicellular organisms such as the human body, growth factors and GFRs are essential during embryogenesis and post‐natal development because they orchestrate cell survival, proliferation, migration and differentiation in all tissues and organs. In the adult body, GFRs are involved in the control of cellular turnover and homeostasis, as well as in tissue repair and regeneration, and their deregulation can lead to cancer. Nerve growth factor (NGF) and epidermal growth factor (EGF) were the first growth factors to be identified, leading to the award of the 1986 Nobel prize in Physiology and Medicine to Stanley Cohen and Rita Levi‐Montalcini.^6^, ^7^ Subsequently, the receptor for EGF (EGFR) was characterized and shown to be a transmembrane receptor with an intracellular tyrosine kinase activity that initiates a cascade of downstream protein phosphorylation when it is engaged by EGF.^6^ As more growth factors were identified, a complex picture of associated GFRs emerged. To date, more than 20 families of GFRs have been described, and, as initially reported for EGFR, most GFRs exhibit a tyrosine kinase activity^8^; the only notable exception being the receptors for members of the transforming growth factor betas (TGFbRs), which exhibit a serine/threonine kinase activity.^9^ Most tyrosine kinase GFRs activate the same set of intracellular kinases, including but not limited to SRC, AKT, PI3PK and ultimately the MAP kinases, all of which translocate into the nucleus to regulate gene expression.^8^ In contrast, the serine/threonine kinase activity of TGFbRs ultimately leads to the activation and nuclear translocation of members of the SMAD transcription factor family.^9^

There are three common characteristics shared by most GFRs: a virtually ubiquitous localization throughout tissues and organs; the existence of multiple GFRs genes/proteins within the same family; and the wide range of cellular effects induced by the same GFRs. This is well illustrated by the receptors for the fibroblast growth factors (FGFRs). There are four different FGFRs (FGFRs1‐4) genes generating seven isoforms. All are expressed in most human cell types and, upon ligand binding, the resultant stimulation of their tyrosine kinases induces a wide range of cellular effects including cell proliferation in epithelial cells and fibroblasts, the migration of endothelial cells, as well as the differentiation of neurons and their subsequent neurite outgrowth. Notably, the activity of FGFRs is also co‐regulated by a class of unbranched glycosaminoglycans, the heparan sulfates (HS), which are abundant on both the cell surface and within the extracellular matrix. HSs make many FGF/FGFR interactions much more efficient by cross‐binding them into trimeric complexes, thus facilitating the activation of FGFRs and their downstream signaling pathways.^10^

## 3 GFR ACTIVITY IN VIRAL ENTRY AND REPLICATION

**FIGURE 1:**
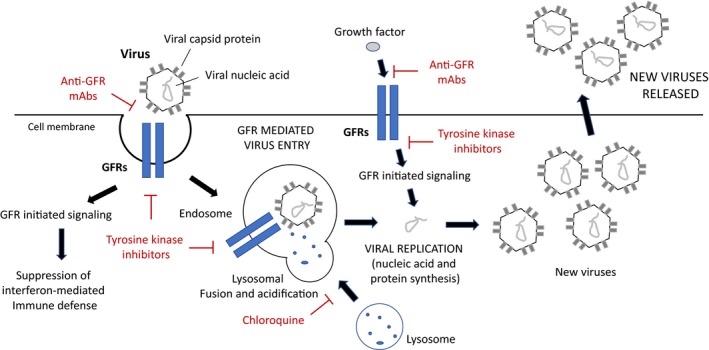
Growth factor receptors (GFRs) involvement in viral entry and replication. Many viruses use GFRs, including Heparan sulfate proteoglycans (HSPGs), as attachment and entry point in human cells. Binding of the virus to extracellular domain of GFRs triggers internalization and the formation of virus‐containing endosome. Fusion with lysosomes induces the destruction of the protein component of the virus and the liberation of the nucleic acid (RNA or DNA depending on virus type). The cell machinery will then replicate and transcribe the viral nucleic acid, ultimately leading to the formation of new viral particles that will be released. Viral entry and replication are also activated by GFR signaling and monoclonal antibodies (mAbs) against GFRs or inhibitors of their tyrosine kinase signaling, which are used in cancer treatment, have been shown to reduce viral replication of many viruses. Noteworthy, GFR activation by viruses has been reported to suppress interferon‐mediated immune defense. Also, chloroquine, an inhibitor of lysosomal activity that is currently in clinical trial for COVID‐19, could potentially interfere with the role of GFRs in viral infection by inhibiting the lysomal degradation of virus‐containing endosomes

The evidence for GFR involvement in viral infections is listed in Table 1, summarized in Figure 1, and further described below.

**TABLE 1:** Representative evidence for the involvement of GFRs in viral infections

Growth factor receptors (GFRs)	Role in viral infections
EGFRs	Hepatitis C virus entry and internalization^12^, ^13^ Hepatitis B virus entry and internalization^14^, ^15^ Gastroenteritis virus entry and internalization^16^, ^17^ Vaccinia virus spread^18^ Suppression of interferon mediated immune defense by many viruses^16^, ^19^ SARS‐CoV ‐induced pulmonary fibrosis^20^
FGFRs	Herpes simplex virus entry and internalization^21^, ^22^ Adeno associate virus entry^25^ Zika virus replication^26^ Influenza virus replication^27^ Dengue virus replication^28^ Epstein Barr virus‐induced cell transformation^29^ MERS‐CoV induced lung cell apoptosis^30^
HSPGs (GFR co‐receptors)	Viruses entry and internalization^23^, ^31^, ^32^ Coronaviruses entry^33^, ^34^, ^35^, ^36^, ^37^
TGFbR	Replication of respiratory syncytial virus^41^, ^42^ MERS‐CoV induced lung fibrosis^30^ SARS‐CoV induced lung fibrosis^43^
TrkA	Influenza virus replication^45^ HIV‐1 virus replication^46^ Rhinoviruses entry^47^, ^48^
EphA2	Hepatitis C virus entry^12^ Epstein‐Barr virus entry^50^, ^51^
PDGFR	Influenza virus entry and internalization^49^
HGFR	Adeno virus entry^44^

Abbreviations: EGFR, epidermal growth factor receptor; FGFRs, fibroblast growth factor receptors; HSPGs, heparan sulfate proteoglycans; TGFbR, transforming growth factor beta receptors; TrkA, tropomyosin‐related tyrosine kinase A (NGF receptors); EphA2, ephrin receptor A2; PDGFR, platelet derived growth factor receptors; HGFR, hepatocyte growth factor receptor; HIV, human immunodeficiency virus; MERS‐CoV, middle east respiratory syndrome coronavirus; SARS‐CoV, severe acute respiratory syndrome coronavirus

### 3.1 EGFR and viral infections

As early as 1978, it was reported that the growth of cytomegalovirus and herpes simplex virus type 1 in human fibroblasts could be altered by EGF,^11^ but the relationship between EGF and viral infection was not understood. As a result, the crosstalk between EGFR and viral protein synthesis was explored, but it was not until 2011 that the EGFR was shown to be the co‐factor responsible for the entry of hepatitis C virus (HCV) into human cells.^12^ This study demonstrated that EGFRs and the EphA2 tyrosine kinase receptors together mediate HCV entry by regulating CD81‐claudin‐1 co‐receptor association, and thus viral glycoprotein‐dependent membrane fusion. This study was also the first to show that tyrosine kinase inhibitors can have substantial antiviral activity.^12^ In turn, the HCV upregulates EGFR activation, which favors further viral entry.^13^ The involvement of EGFRs with virus internalization and transport to the endosomal network was further confirmed in the case of hepatitis B virus (HBV)^14^, ^15^ as well as for gastroenteritis viruses.^16^, ^17^ The vaccinia virus (VACV) has also been shown to co‐opt EGFR signaling to enhance virus spread through the stimulation of cell motility that contributes to the rapid spread of infection.^18^ Significantly, many respiratory viruses have been shown to induce EGFR activation, leading to suppressed interferon regulating factor‐1 (IRF1)‐dependent interferon λ and thus lowered antiviral defense in airway epithelium.^16^, ^19^ Thus, EGFRs are not only portals for virus entry, but are also involved in the suppression of the immune response of the host.

The overactivation of EGFRs could also result in other long‐term issues for patients affected by respiratory viruses, as was shown with the severe acute respiratory syndrome (SARS) coronavirus; EGRF overactivation leads to increased pulmonary fibrosis.^20^ It is therefore tempting to hypothesize that EGFR inhibition might also reduce the risk of further pulmonary fibrotic damage after coronavirus infection.

### 3.2 FGFRs and viral infection

The first study demonstrating a role for FGFRs in viral infection reported that they were a portal of cellular entry for the herpes simplex virus type 1, and presumed it was through the binding of FGF to the viral particle and presentation to the receptors.^21^, ^22^ However, a subsequent and contradictory study found that FGFRs were not required for herpes simplex virus type 1 infection,^23^ but that FGF‐binding heparan sulfates (HS) on the cell surface and in the pericellular space were involved instead.^24^ It is now well established that HS is essential to the interaction of HS‐binding FGFs with FGFR, because it creates a trimolecular FGF‐HS‐FGFR complex that subsequently triggers FGFR activation^9^ and such a scenario resolved the initially conflicting results. FGFR1 was then shown to be a co‐receptor for infection by adeno‐associated virus 2.^25^ FGFR signaling also accompanies Zika virus infection in human fetal brain.^26^ Using an integrative systems approach based on genome‐wide RNA interference screening, FGFRs were found to be co‐factors required for early‐stage influenza virus replication.^27^ A reciprocal effect of FGFR signaling has also been reported for the dengue virus, with the inhibition of FGFRs reducing virus RNA replication while increasing viral particle production.^28^

Similar to the case of EGFRs, FGFR activation may trigger long‐term consequences for virus infected patients. The activation of the FGFR1 signaling pathway by Epstein‐Barr virus‐encoded LMP1 promotes aerobic glycolysis and ultimately the cancerous transformation of human nasopharyngeal epithelial cells.^29^ Taken together, as FGFRs and heparan sulfates participate in viral infection, the data strongly suggest they could potentially be targeted to inhibit virus development. Supporting this contention, the middle east respiratory syndrome coronavirus (MERS‐CoV), a close relative to SARS‐CoV‐2, is known to induce lung cell apoptosis that results in major lung damage by upregulating FGF‐2.^30^

### 3.3 HS proteoglycans (HSPGs) and viral infections

As well as facilitating FGFR activation, the HS complexed into proteoglycan complexes to form HSPGs represent a separate entry point for viruses into human cells. This was first shown for the herpes simplex virus, whose initial interaction with cells is via binding to cell surface HSPGs.^31^, ^32^ There is now abundant evidence that coronaviruses use HSPGs to attach to the plasma membrane before internalization, with the first demonstrations provided for a murine coronavirus^33^ and an avian bronchitis coronavirus.^34^ Similarly, the human coronavirus NL63 also utilizes such HSPGs for attachment to target nasopharyngeal cells.^35^, ^36^ Interestingly, a SARS virus variant has also been shown to utilize HSPG for cell entry, which can be competitively inhibited by lactoferrin.^37^ Together, these data on coronaviruses suggest that SARS‐Cov2 is also likely to bind to HSPG; preliminary data have recently been released that reinforce this hypothesis.^38^ In this study, the authors used surface plasmon resonance and circular dichroism to measure the interaction between the SARS‐CoV‐2 Spike S1 protein receptor binding domain (SARS‐CoV‐2 S1 RBD) and heparin.^38^ The data strongly suggested an interaction between the recombinant surface receptor binding domain of the virus and HS. The therapeutic targeting of HSPGs thus appears a relatively straightforward way to inhibit the infectivity of SARS‐Cov2.

### 3.4 TGFbRs and viral infections

TGFbRs have not been reported as a cellular entry point for viruses; however, TGFbR stimulation appears to regulate viral replication. It was initially shown that TGFbR stimulation resulted in the suppression of the human immunodeficiency virus (HIV) expression and replication.^39^ The same inhibitory effect of TGFbRs was found with HCV.^40^ In contrast, TGFbRs enhance respiratory syncytial virus (RSV) replication in epithelial cells^41^ via reduction of hepatocyte nuclear factor‐4a expression.^42^

Of note, the MERS coronavirus induces apoptosis in lung cells by upregulating SMAD7, a key protein involved in TGFbR signaling.^30^ The nucleocapsid of the coronavirus SARS‐Cov interacts with SMAD3 to modulate TGFbR signaling, resulting in the promotion of lung fibrosis^43^ and possible long‐term deleterious effects for patients.

### 3.5 Other GFRs in viral infections

There is less information on the role of other classes of GFRs in virus infection. The hepatocyte growth factor receptor (HGFR) is a known co‐receptor for cell entry of adeno‐associated virus type 2 infection.^44^ Pharmacological inhibition of the NGF receptor TrkA has been shown to suppress influenza virus replication.^45^ NGF stimulates HIV‐1 replication in primary macrophages by signaling through TrKA, with the involvement of reticular calcium, protein kinase C, extracellular signal‐regulated kinase, p38 kinase, and nuclear factor‐κB.^46^ NGF‐induced enhancement of HIV‐1 replication occurs during the late events of the HIV‐1‐replicative cycle, with a concomitant increase in viral transcription and production.^46^ Similarly, it has been shown that human rhinoviruses (HRV) upregulate the NGF‐TrkA pathway in airway epithelial cells, which in turn amplifies viral replication by increasing HRV entry via ICAM‐1 receptors and by limiting apoptosis of nasopharyngeal cells.^47^, ^48^ Recent evidence suggests that the influenza virus uses the GM3‐enhanced platelet‐derived growth factor receptor beta (PDGFRb) signaling pathway for cell penetration^49^; this study also reported that the tyrosine kinase inhibitor Ki8751 disrupts the endocytotic process of influenza A and B viruses induced by PDGFRb.^49^ Importantly, the tyrosine kinase ephrin receptor A2 has been reported to participate in HCV entry^12^ and is also an epithelial receptor for Epstein‐Barr virus (EBV) entry into epithelial cells, thus increasing the risk of malignancy.^50^, ^51^

## 4 CURRENT DRUGS TARGETING GFRS AND THEIR POTENTIAL USE IN VIRAL INFECTION

The evidence described above points to the extensive involvement of GFRs in the processes of virus entry and replication. It is not only that some viruses can use GFRs as attachment receptor for cellular internalization, but it also appears that their downstream tyrosine kinase activity promotes virus replication. Importantly there is a range of drugs that are already in use in oncology to target GFRs and their tyrosine kinase activities in the treatment of cancer patients. The currently used drugs can be classified into two categories: blocking monoclonal antibodies (mAbs) and tyrosine kinase inhibitors. In addition, clinical heparin can also regulate GFR activation and virus entry.

### 4.1 Blocking mAbs against GFRs

These have been developed specifically to bind to the extracellular domain of GFRs to block ligand binding and thereby inhibit receptor dimerization, autophosphorylation and downstream signaling.^52^ Trastuzumab (also called Herceptin), a humanized mAb, was the first GFR‐targeted drug to be introduced into oncology and is routinely used to treat breast cancers overexpressing the EGFR family member HER2 (human epidermal growth factor receptor 2, also called ErbB2).^53^ Patients overexpressing HER2 classically have a poor prognosis, but since the introduction of Trastuzumab, in combination with chemotherapy, this situation has completely changed; HER2‐positive patients now have a survival rate similar to median breast cancer survival rates.^53^ In terms of side effects, when compared to chemotherapy, Trastuzumab is well tolerated. Other mAbs against EGFR, such as Cetuximab and Panitumumab, are also used clinically, particularly for colorectal cancer, and head and neck cancers. Given the reported involvement of EGFRs for cellular entry and replication of many viruses, mAbs against EGFRs are clearly worthy of further testing, not only against newly emerging viruses, but also against SARS‐CoV‐2 itself.

### 4.2 Pharmacological inhibitors of the tyrosine kinase activity of GFRs

Tyrosine kinase inhibitors (TKIs) bind to ATP‐binding pockets located within the intracellular catalytic kinase domain of tyrosine kinase GFRs, so blocking the activation of downstream signaling. They are also frontline targeted therapies for the treatment of several cancer types. They thus hold potential interest for drug repurposing against viruses. TKIs were first developed to target EGFR family members and they were the first to enter clinical use in oncology.^54^ First‐generation EGFR TKIs, such as Gefitinib and Erlotinib, reversibly bind to EGFRs, thereby improving the survival of patients with lung cancer which harbor EGFR‐activating mutations.^55^ Second‐generation EGFR TKIs, such as Afatinib and Dacomitinib, irreversibly bind to such receptors and show an increased cellular potency against EGFR oncogenic variants.^56^ Several anti‐GFR TKIs, including those targeting more specifically HER2, TrkA, and PDGFR, have already been shown to block multiple steps of the replication of both influenza virus^45^ and dengue virus.^57^ Evidently, such encouraging results should prompt further testing against emerging coronavirus species.

### 4.3 Heparin

HSs found on the cell surface and as part of the extracellular matrix are essential to the activation of several GFRs, mostly by virtue of their facilitation of growth factor binding. Structurally very close to the native ECM glycosaminoglycan HS is the variant heparin, one of the most widely used drugs in the world. Heparin and its derivatives are clinically approved as anticoagulants/anti‐thrombotics, with well‐known bioavailability albeit with a tight safety window. Not all heparin derivatives have anti‐coagulant activity. Importantly, heparin has been shown to have a broad spectrum of activity against many viruses, including Zika^58^ and HIV‐1,^59^ presumably through a competitively inhibitive process between it and cellular HS, whereby an excess of heparin prevents a virus from binding to cell surface HS. Because coronaviruses have been shown to bind to HS to enter target cells,^35^, ^36^ it can be hypothesized that heparin should act to prevent cell attachment of SARS‐CoV‐2. Preliminary data have indicated that indeed heparin does bind to the surface protein (spike) S1 of this virus^38^ and may therefore be therapeutic for COVID‐19.

## 5 CONCLUSION: ANTI‐GFR DRUG REPURPOSING FOR EMERGING VIRAL INFECTIONS INCLUDING COVID‐19

The literature described herein emphasizes the pivotal involvement of GFRs, and co‐receptors such as HSPGs, in viral entry and replication. In the face of the current lack of a vaccine or other effective therapies for the treatment of emerging viral diseases such as COVID‐19, with their dramatic health and economic consequences, the targeting of GFRs using existing oncologic drugs represents an intriguing opportunity. Given the rapidity of COVID‐19 spread throughout the world, drug repurposing offers a fast track for the introduction of therapies using known clinically approved molecules. The value of drug repurposing to fight SARS‐CoV‐2 is well illustrated with chloroquine/hydroxychloroquine, an inexpensive drug with low toxicity that has been used for more than 60 years for the treatment of malaria. It has shown promising effects in reducing the viral load and the duration of infection in initial clinical trials involving patients with COVID‐19.^60^, ^61^ Interestingly, chloroquine is known to inhibit the activation of mitogen‐activated protein kinases (MAPKs), which are activated by GFRs.^62^ For the HCoV‐229 coronavirus, chloroquine‐induced virus inhibition occurs via its inhibition of MAPK,^63^ suggesting that MAPK‐activating GFRs are involved. Although at this stage it has not yet been demonstrated that GFRs are involved in SARS‐CoV‐2 cell entry and replication, the current knowledge about the role of GFRs in other viral infections, including other coronaviruses, constitutes a rational basis to begin testing the value of repurposed anti‐GFRs drugs against emerging viral diseases, and COVID‐19 in particular.

## CONFLICT OF INTEREST

The authors declare no conflicts of interest.

## References

[1] Del Rio C , Malani PN . COVID‐19—new insights on a rapidly changing epidemic. JAMA. 2020 [Epub ahead of print]. 10.1001/jama.2020.3072 32108857

[2] Walls AC , Park Y‐J , Tortorici MA , Wall A , McGuire AT , Veesler D . Structure, function, and antigenicity of the SARS‐CoV‐2 spike glycoprotein. Cell. 2020 [Epub ahead of print]. 10.1016/j.cell.2020.02.058 PMC7102599 32155444

[3] Hoffmann M , Kleine‐Weber H , Schroeder S , et al. SARS‐CoV‐2 cell entry depends on ACE2 and TMPRSS2 and is blocked by a clinically proven protease inhibitor. Cell. 2020 [Epub ahead of print]. 10.1016/j.cell.2020.02.052 PMC7102627 32142651

[4] Letko M , Marzi A , Munster V . Functional assessment of cell entry and receptor usage for SARS‐CoV‐2 and other lineage B betacoronaviruses. Nat Microbiol. 2020;5(4):562‐569. 32094589 10.1038/s41564-020-0688-y PMC7095430

[5] Yan R , Zhang Y , Li Y , Xia L , Guo Y , Zhou Q . Structural basis for the recognition of the SARS‐CoV‐2 by full‐length human ACE2. Science. 2020;6485:eabb2762. 10.1126/science.abb2762 PMC7164635 32132184

[6] Cohen S . Origins of growth factors: NGF and EGF. J Biol Chem. 2008;283:33793‐33797. 18697735 10.1074/jbc.X800008200 PMC2662208

[7] Levi‐Montalcini R . From a home‐made laboratory to the Nobel Prize: an interview with Rita Levi Montalcini. Int J Dev Biol. 2000;44:563‐566. 11061418

[8] Wheeler DL , Yarden Y . Receptor Tyrosine Kinases: Family and Subfamilies. Totowa, NJ: Humana Press; 2015.

[9] Batlle E , Massagué J . Transforming growth factor‐β signaling in immunity and cancer. Immunity. 2019;50:924‐940. 30995507 10.1016/j.immuni.2019.03.024 PMC7507121

[10] Nurcombe V , Ling L , Hondermarck H , Cool SM , Smith RAA . Bringing heparan sulfate glycomics together with proteomics for the design of novel therapeutics: a historical perspective. Proteomics. 2019;19:e1800466. 31197945 10.1002/pmic.201800466

[11] Knox GE , Reynolds DW , Cohen S , Alford CA . Alteration of the growth of cytomegalovirus and herpes simplex virus type 1 by epidermal growth factor, a contaminant of crude human chorionic gonadotropin preparations. J Clin Invest. 1978;61:1635‐1644. 207740 10.1172/JCI109084 PMC372690

[12] Lupberger J , Zeisel MB , Xiao F , et al. EGFR and EphA2 are host factors for hepatitis C virus entry and possible targets for antiviral therapy. Nat Med. 2011;17:589‐595. 21516087 10.1038/nm.2341 PMC3938446

[13] Diao J , Pantua H , Ngu H , et al. Hepatitis C virus induces epidermal growth factor receptor activation via CD81 binding for viral internalization and entry. J Virol. 2012;86:10935‐10949. 22855500 10.1128/JVI.00750-12 PMC3457153

[14] Iwamoto M , Saso W , Sugiyama R , et al. Epidermal growth factor receptor is a host‐entry cofactor triggering hepatitis B virus internalization. Proc Natl Acad Sci USA. 2019;116:8487‐8492. 30952782 10.1073/pnas.1811064116 PMC6486715

[15] Iwamoto M , Saso W , Nishioka K , et al. The machinery for endocytosis of epidermal growth factor receptor coordinates the transport of incoming hepatitis B virus to the endosomal network. J Biol Chem. 2020;295:800‐807. 31836663 10.1074/jbc.AC119.010366 PMC6970923

[16] Yang L , Xu J , Guo L , et al. Porcine epidemic diarrhea virus‐induced epidermal growth factor receptor activation impairs the antiviral activity of type I interferon. J Virol. 2018;92(8):e02095‐e2117. 29386292 10.1128/JVI.02095-17 PMC5874413

[17] Hu W , Zhang S , Shen Y , Yang Q . Epidermal growth factor receptor is a co‐factor for transmissible gastroenteritis virus entry. Virology. 2018;521:33‐43. 29879540 10.1016/j.virol.2018.05.009 PMC7112045

[18] Beerli C , Yakimovich A , Kilcher S , et al. Vaccinia virus hijacks EGFR signalling to enhance virus spread through rapid and directed infected cell motility. Nat Microbiol. 2019;4:216‐225. 30420785 10.1038/s41564-018-0288-2 PMC6354922

[19] Ueki IF , Min‐Oo G , Kalinowski A , et al. Respiratory virus‐induced EGFR activation suppresses IRF1‐dependent interferon λ and antiviral defense in airway epithelium. J Exp Med. 2013;210:1929‐1936. 23999497 10.1084/jem.20121401 PMC3782052

[20] Venkataraman T , Coleman CM , Frieman MB . Overactive epidermal growth factor receptor signaling leads to increased fibrosis after severe acute respiratory syndrome coronavirus infection. J Virol. 2017;91(12):e00182‐e217. 28404843 10.1128/JVI.00182-17 PMC5446658

[21] Kaner RJ , Baird A , Mansukhani A , et al. Fibroblast growth factor receptor is a portal of cellular entry for herpes simplex virus type 1. Science. 1990;248:1410‐1413. 2162560 10.1126/science.2162560

[22] Baird A , Florkiewicz RZ , Maher PA , Kaner RJ , Hajjar DP . Mediation of virion penetration into vascular cells by association of basic fibroblast growth factor with herpes simplex virus type 1. Nature. 1990;348:344‐346. 2174511 10.1038/348344a0

[23] Lycke E , Johansson M , Svennerholm B , Lindahl U . Binding of herpes simplex virus to cellular heparan sulphate, an initial step in the adsorption process. J Gen Virol. 1991;72(Pt 5):1131‐1137. 1851813 10.1099/0022-1317-72-5-1131

[24] Mirda DP , Navarro D , Paz P , Lee PL , Pereira L , Williams LT . The fibroblast growth factor receptor is not required for herpes simplex virus type 1 infection. J Virol. 1992;66:448‐457. 1309254 10.1128/jvi.66.1.448-457.1992 PMC238305

[25] Qing K , Mah C , Hansen J , Zhou S , Dwarki V , Srivastava A . Human fibroblast growth factor receptor 1 is a co‐receptor for infection by adeno‐associated virus 2. Nat Med. 1999;5:71‐77. 9883842 10.1038/4758

[26] Limonta D , Jovel J , Kumar A , et al. Fibroblast growth factor 2 enhances zika virus infection in human fetal brain. J Infect Dis. 2019;220:1377‐1387. 30799482 10.1093/infdis/jiz073 PMC6743838

[27] König R , Stertz S , Zhou Y , et al. Human host factors required for influenza virus replication. Nature. 2010;463:813‐817. 20027183 10.1038/nature08699 PMC2862546

[28] Cortese M , Kumar A , Matula P , et al. Reciprocal effects of fibroblast growth factor receptor signaling on dengue virus replication and virion production. Cell Rep. 2019;27(2579–2592):e6. 10.1016/j.celrep.2019.04.105 31141684

[29] Lo AK , Dawson CW , Young LS , Ko CW , Hau PM , Lo KW . Activation of the FGFR1 signalling pathway by the Epstein‐Barr virus‐encoded LMP1 promotes aerobic glycolysis and transformation of human nasopharyngeal epithelial cells. J Pathol. 2015;237:238‐248. 26096068 10.1002/path.4575

[30] Yeung ML , Yao Y , Jia L , et al. MERS coronavirus induces apoptosis in kidney and lung by upregulating Smad7 and FGF2. Nat Microbiol. 2016;1:16004. 27572168 10.1038/nmicrobiol.2016.4 PMC7097571

[31] WuDunn D , Spear PG . Initial interaction of herpes simplex virus with cells is binding to heparan sulfate. J Virol. 1989;63:52‐58. 2535752 10.1128/jvi.63.1.52-58.1989 PMC247656

[32] Shieh MT , WuDunn D , Montgomery RI , Esko JD , Spear PG . Cell surface receptors for herpes simplex virus are heparan sulfate proteoglycans. J Cell Biol. 1992;116:1273‐1281. 1310996 10.1083/jcb.116.5.1273 PMC2289355

[33] de Haan CA , Li Z , te Lintelo E , Bosch BJ , Haijema BJ , Rottier PJ . Murine coronavirus with an extended host range uses heparan sulfate as an entry receptor. J Virol. 2005;79:14451‐14456. 16254381 10.1128/JVI.79.22.14451-14456.2005 PMC1280238

[34] Madu IG , Chu VC , Lee H , Regan AD , Bauman BE , Whittaker GR . Heparan sulfate is a selective attachment factor for the avian coronavirus infectious bronchitis virus Beaudette. Avian Dis. 2007;51:45‐51. 17461266 10.1637/0005-2086(2007)051[0045:HSIASA]2.0.CO;2

[35] Milewska A , Zarebski M , Nowak P , Stozek K , Potempa J , Pyrc K . Human coronavirus NL63 utilizes heparan sulfate proteoglycans for attachment to target cells. J Virol. 2014;88:13221‐13230. 25187545 10.1128/JVI.02078-14 PMC4249106

[36] Milewska A , Nowak P , Owczarek K , et al. Entry of human coronavirus NL63 into the cell. J Virol. 2018;92(3):e01933‐17. 29142129 10.1128/JVI.01933-17 PMC5774871

[37] Lang J , Yang N , Deng J , et al. Inhibition of SARS pseudovirus cell entry by lactoferrin binding to heparan sulfate proteoglycans. PLoS ONE. 2011;6:e23710. 21887302 10.1371/journal.pone.0023710 PMC3161750

[38] Mycroft‐West C , Dunhao SU , Elli S , et al. coronavirus (SARS‐CoV‐2) surface protein (Spike) S1 receptor binding domain undergoes conformational change upon heparin binding. BioRxiv; 2019 10.1101/2020.02.29.971093

[39] Poli G , Kinter AL , Justement JS , Bressler P , Kehrl JH , Fauci AS . Transforming growth factor beta suppresses human immunodeficiency virus expression and replication in infected cells of the monocyte/macrophage lineage. J Exp Med. 1991;173:589‐597. 1705278 10.1084/jem.173.3.589 PMC2118806

[40] Murata T , Ohshima T , Yamaji M , et al. Suppression of hepatitis C virus replicon by TGF‐beta. Virology. 2005;331:407‐417. 15629783 10.1016/j.virol.2004.10.036

[41] McCann KL , Imani F . Transforming growth factor beta enhances respiratory syncytial virus replication and tumor necrosis factor alpha induction in human epithelial cells. J Virol. 2007;81:2880‐2886. 17202225 10.1128/JVI.02583-06 PMC1866016

[42] Hong MH , Chou YC , Wu YC , et al. Transforming growth factor‐β1 suppresses hepatitis B virus replication by the reduction of hepatocyte nuclear factor‐4α expression. PLoS ONE. 2012;7:e30360. 22276183 10.1371/journal.pone.0030360 PMC3262823

[43] Zhao X , Nicholls JM , Chen YG . Severe acute respiratory syndrome‐associated coronavirus nucleocapsid protein interacts with Smad3 and modulates transforming growth factor‐beta signaling. J Biol Chem. 2008;283:3272‐3280. 18055455 10.1074/jbc.M708033200 PMC8740907

[44] Kashiwakura Y , Tamayose K , Iwabuchi K , et al. Hepatocyte growth factor receptor is a coreceptor for adeno‐associated virus type 2 infection. J Virol. 2005;79:609‐614. 15596854 10.1128/JVI.79.1.609-614.2005 PMC538679

[45] Kumar N , Liang Y , Parslow TG , Liang Y . Receptor tyrosine kinase inhibitors block multiple steps of influenza a virus replication. J Virol. 2011;85:2818‐2827. 21209112 10.1128/JVI.01969-10 PMC3067926

[46] Souza TM , Rodrigues DQ , Passaes CP , et al. The nerve growth factor reduces APOBEC3G synthesis and enhances HIV‐1 transcription and replication in human primary macrophages. Blood. 2011;117:2944‐2952. 21217078 10.1182/blood-2010-05-287193

[47] Othumpangat S , Gibson LF , Samsell L , Piedimonte G . NGF is an essential survival factor for bronchial epithelial cells during respiratory syncytial virus infection. PLoS ONE. 2009;4:e6444. 19649262 10.1371/journal.pone.0006444 PMC2715860

[48] Othumpangat S , Regier M , Piedimonte G . Nerve growth factor modulates human rhinovirus infection in airway epithelial cells by controlling ICAM‐1 expression. Am J Physiol Lung Cell Mol Physiol. 2012;302(10):L1057‐L1066. 22427528 10.1152/ajplung.00365.2011 PMC3362265

[49] Vrijens P , Noppen S , Boogaerts T , et al. Influenza virus entry via the GM3 ganglioside‐mediated platelet‐derived growth factor receptor β signalling pathway. J Gen Virol. 2019;100:583‐601. 30762518 10.1099/jgv.0.001235

[50] Chen J , Sathiyamoorthy K , Zhang X , et al. Ephrin receptor A2 is a functional entry receptor for Epstein‐Barr virus. Nat Microbiol. 2018;3:172‐180. 29292384 10.1038/s41564-017-0081-7 PMC5972547

[51] Zhang H , Li Y , Wang HB , et al. Ephrin receptor A2 is an epithelial cell receptor for Epstein‐Barr virus entry. Nat Microbiol. 2018;3:1‐8. 10.1038/s41564-017-0080-8 29292383

[52] Weiner LM , Surana R , Wang S . Monoclonal antibodies: versatile platforms for cancer immunotherapy. Nat Rev Immunol. 2010;10:317‐327. 20414205 10.1038/nri2744 PMC3508064

[53] Maximiano S , Magalhães P , Guerreiro MP , Morgado M . Trastuzumab in the treatment of breast cancer. BioDrugs. 2016;30:75‐86. 26892619 10.1007/s40259-016-0162-9

[54] Ciardiello F , Tortora G . EGFR antagonists in cancer treatment. N Engl J Med. 2008;358:1160‐1174. 18337605 10.1056/NEJMra0707704

[55] Sim EHA , Yang IA , Wood‐Baker R , Bowman RV , Fong KM . Gefitinib for advanced non‐small cell lung cancer. Cochrane Database Syst Rev. 2018;1:CD006847. 29336009 10.1002/14651858.CD006847.pub2 PMC6491254

[56] Deeks ED , Keating GM . Afatinib in advanced NSCLC: a profile of its use. Drugs Ther Perspect. 2018;34:89‐98. 29540977 10.1007/s40267-018-0482-6 PMC5840214

[57] Duran A , Valero N , Mosquera J , Fuenmayor E , Alvarez‐Mon M . Gefitinib and pyrrolidine dithiocarbamate decrease viral replication and cytokine production in dengue virus infected human monocyte cultures. Life Sci. 2017;191:180‐185. 29055802 10.1016/j.lfs.2017.10.027

[58] Ghezzi S , Cooper L , Rubio A , et al. Heparin prevents Zika virus induced‐cytopathic effects in human neural progenitor cells. Antiviral Res. 2017;140:13‐17. 28063994 10.1016/j.antiviral.2016.12.023 PMC7113768

[59] Harrop HA , Rider CC . Heparin and its derivatives bind to HIV‐1 recombinant envelope glycoproteins, rather than to recombinant HIV‐1 receptor, CD4. Glycobiology. 1998;8:131‐137. 9451022 10.1093/glycob/8.2.131

[60] Yao X , Ye F , Zhang M , et al. In vitro antiviral activity and projection of optimized dosing design of hydroxychloroquine for the treatment of severe acute respiratory syndrome coronavirus 2 (SARS‐CoV‐2). Clin Infect Dis. 2020 [Epub ahead of print]. 10.1093/cid/ciaa237 PMC7108130 32150618

[61] Devaux CA , Rolain JM , Colson P , Raoult D . New insights on the antiviral effects of chloroquine against coronavirus: what to expect for COVID‐19? Int J Ant Agents. 2020 [Epub ahead of print]. 10.1016/j.ijantimicag.2020.105938 PMC7118659 32171740

[62] Steiz M , Valbracht J , Quach J , Lotz M . Gold sodium thiomalate and chloroquine inhibit cytokine production in monocytic THP‐1 cells through distinct transcriptional and posttranslational mechanisms. J Clin Immunol. 2003;23:477‐484. 15031635 10.1023/b:joci.0000010424.41475.17

[63] Kono M , Tatsumi K , Imai AM , Saito K , Kuriyama T , Shirasawa H . Inhibition of human coronavirus 229E infection in human epithelial lung cells (L132) by chloroquine: involvement of p38 MAPK and ERK. Antiviral Res. 2008;77:150‐152. 18055026 10.1016/j.antiviral.2007.10.011 PMC7114149

